# Explanatory models and animal health-seeking behavior for East Coast fever in rural Kenya: an ethnographic study

**DOI:** 10.3389/fvets.2024.1400467

**Published:** 2024-09-03

**Authors:** Ann W. Muthiru, Josphat Muema, Nyamai Mutono, S. M. Thumbi, Salome A. Bukachi

**Affiliations:** ^1^Department of Anthropology, Gender and African Studies, University of Nairobi, Nairobi, Kenya; ^2^Feed the Future Innovation Lab for Animal Health, Washington State University, Pullman, WA, United States; ^3^Centre for Epidemiological Modelling and Analysis, University of Nairobi, Nairobi, Kenya; ^4^Washington State University Global Health Program – Kenya, Nairobi, Kenya; ^5^Paul G. Allen School for Global Health, Washington State University, Pullman, WA, United States

**Keywords:** rural Kenya, east coast fever, explanatory models, infection treatment method, health-seeking behaviorethnography

## Abstract

**Introduction:**

Explanatory models of disease focus on individuals’ and groups’ understandings of diseases, revealing a disconnect between livestock keepers and animal health providers. Animal health providers rely on models grounded in their veterinary training and experience. At the same time, livestock keepers may construct models based on traditional knowledge and their lived experience with East Coast fever in their cattle herds. To better understand East Coast fever and develop more efficient management strategies, this ethnographic study used the explanatory models’ framework to provide a structured way for comprehending and contrasting different beliefs and understandings of East Coast fever as perceived by the livestock keepers across the different livestock production systems.

**Method:**

Multiple data collection methods were employed, including unstructured observations, 30 in-depth interviews (IDIs), 18 focus group discussions (FGDs), and 25 key informant interviews (KIIs).

**Results:**

Adult cattle, calves and sheep were perceived as susceptible to East Coast fever. However, there were varying perceptions of livestock susceptible to East Coast fever in the different livestock production systems. East Coast fever was attributed to multiple factors, including ticks, tsetse flies, mosquitos, birds, stagnant, dirty, or contaminated water, and livestock-wildlife interactions. However, some aspects were specific to the production system. Livestock keepers classified diseases based on observable signs, grouping diseases with similar signs under the same classification. Moreover, livestock keepers described different forms of East Coast fever ranging from treatable to fatal, which could be distinguished by the signs they presented. Self-treatment with drugs from the local agro-vet shops was the initial course of action during suspected cases of East Coast fever. Animal health practitioners were the last resort if self-treatment did not produce the desired outcome. Livestock keepers perceived avoidance of stagnant or contaminated water, tick control, and fencing as effective control measures for East Coast fever in their livestock herd. Very few livestock keepers were aware of an East Coast fever vaccine.

**Discussion:**

Mechanistic explanations hold little significance in controlling East Coast fever. Instead, understanding and addressing livestock keepers’ beliefs regarding ECF is crucial for promoting behaviors that support interventions across different livestock production systems.

## 1 Introduction

Communities in many parts of Africa rely on livestock for their socio-economic well-being and food security (1, 2). However, livestock diseases pose severe challenges to livestock keepers’ livelihoods, threatening the sustainability of these crucial livestock resources. East Coast fever (ECF), a fatal endemic bovine disease, is one of many livestock diseases that negatively impacts the livelihoods of pastoralists through a decrease in milk production, reduced draft power, poor condition of the cattle associated with lower value at the market, and slaughter as well as lower fertility rates (3). East Coast fever (ECF) is a cattle tickborne disease (TBD) caused by a protozoan parasite called *Theileria parva*. The parasite is transmitted by a three-host tick called *Rhipicephalus appendiculatus,* which has dropped from an infected cattle during the preceding stages of the life cycle (4). East Coast fever is endemic to eastern, central and southern parts of Africa. It has been reported in Kenya, Uganda, Tanzania, Burundi, Rwanda, Mozambique, Malawi, the Democratic Republic of Congo (DRC), southern Sudan, Zimbabwe and Zambia (5). East Coast fever (ECF) threatens about 28 million cattle and kills over 1 million cattle annually in these endemic areas; small-scale farmers suffer the most from these losses due to their high dependence on cattle for their livelihoods and food security (6).

In Kenya, *Theileria parva* infection presents a significant threat to the livestock sector, manifesting in two significant ways: the disease’s economic toll from cattle morbidity and mortality, leading to production losses across all systems. The financial burden is further exacerbated by the substantial costs of tick control measures. For example, the application of acaricides, which is the main method of tick control, has been estimated to cost between US$6 and US$36 per adult animal in East African countries including Kenya (6). Furthermore, the disease hinders the introduction of more productive exotic breeds that are susceptible to ECF, thereby significantly impeding the development of the livestock sector (7).

Despite significant challenges, cattle can be protected against ECF by an immunization process called the Infection Treatment Method (8). This involves treating the cattle by administering a long-acting antibiotic (oxytetracycline) alongside providing it with a dosage of *Theileria parva* parasites. As a result, the cattle develop immunity to related or similar parasites throughout their lives (8). However, the use of long-acting antibiotics raises concerns about the development of antibiotic resistance. Although this combination is effective, it is crucial to acknowledge the possibility of resistance developing if the antibiotics are not used appropriately. With its potential to significantly increase livestock productivity, the ITM could positively impact livelihoods, food security, and the realization of the United Nations Sustainable Development Goals (SDGs)1,2, 3 and 5 on no poverty, zero hunger as well as good health and well-being (9). However, the main obstacle lies in the limited use of the ITM among small-scale farmers, which presents a significant barrier to realizing the full potential of this vaccine and, therefore, demands an immediate response to guarantee widespread adoption by livestock keepers.

Prior research on ITM adoption has focused on socio-economic factors such as off-farm occupation, herd size, on-farm tick control method, and gender-based adoption rate differences (10, 11). Only a few studies reported on livestock keepers’ knowledge attitudes and practices (KAP) on ECF and their influence on ECF control measures (12, 13). However, while KAP surveys have been used, they have been limited in capturing the different types of knowledge within different communities or cultures. Furthermore, attitudes are difficult to capture within the framework of surveys as they are subjective and context-specific. These limitations render it challenging to make definitive statements about actual practices or conduct an in-depth analysis of underlying context-related factors, impacting the credibility of the findings (14, 15). Moreover, KAP surveys lean only towards the biomedical explanatory model of health and disease.

Health and illness considerations are universal, transcending cultural and social divides, and have been woven into the foundation of human existence. Using material resources, concepts, and cultural components, every social group organizes itself collectively (16) to understand health-related events or barriers and develop suitable responses. These personal or collective responses reflect the diverse and complex strategies developed by these communities. These approaches demonstrate the variety of ways that human societies adapt to, interpret, and manage health challenges within their specific socio-cultural frameworks. Building upon this discourse, Arthur Kleinman’s explanatory models (EMs), model focus on how people’s attitudes about health and illness are shaped by their social and cultural background, which influences how people perceive signs and health behavior, both preventative and treatment-seeking. In Kleinman’s classic formulation, EMs are constructed from five key elements: The definition of the illness; Etiology; Onset of signs; Course of sickness including its severity and likely progression; Treatment and measures likely to be efficacious (17). By taking into account these aspects, the model provides a thorough perspective through which to view the culturally established health response mechanisms.

According to Kleinman’s (17) theory of EMs, individuals and groups can have various notions of health and disease. Explanatory Models by animal health practitioners are predominantly biomedical, focusing on the biological and physical aspects of disease origin and causation. However, distinct EMs may apply to individuals or groups experiencing illness; social and cultural contexts and prior experiences influence these. In some instances, although the biomedical model may influence the explanations of individuals or groups, the importance placed on particular aspects may vary between the animal health practitioners or individuals or groups (18, 19). Many studies on livestock and zoonotic diseases from non-Western cultures have been shown to have a holistic concept of health and diseases that entail a supernatural aspect of disease causation. In Tanzania, a study revealed that among agro-pastoralists, brucellosis signs in livestock, such as abortion, stillbirth, and infertility, were attributed to supernatural causes (19). In Northern Kenya, another study revealed that livestock keepers believed that Rift Valley fever (RVF) was a curse that required the intervention of a spiritual leader and ritual slaughter for cleansing of the victim (20).

Studies have also shown that the type of EM held by individuals or groups influences their response to health messages and health behaviors such as prevention and seeking treatment. Further, EMs affect the type of healer or doctor to visit and the treatment course that will be taken (19, 20).

Therefore, understanding livestock keepers’ explanatory model is important for developing culturally targeted health messages and intervention strategies that incorporate livestock keepers’ EMs. This study aimed to apply the explanatory models’ framework to bridge the gap between biomedical and experiential understanding of ECF, particularly in diverse livestock production systems.

## 2 Materials and methods

### 2.1 Study area and population

This study was conducted in the Narok South sub-county in Narok County, situated in the Great Rift Valley in southern Kenya, which borders the Republic of Tanzania. The basic characteristics of the Narok area have been characterized by agroecological zones (AEZs) II, III (sub-humid to semi-humid), IV (semi-humid to semiarid), and V, VI, and VII (semiarid to very arid). The climatic conditions are favorable for the tick vectors of tick-borne diseases (TBDs), namely, *Rhipicephalus appendiculatus* for ECF (7). Therefore, unless tick management measures are implemented or there is endemic stability in the area, cattle in this region are constantly at risk of developing severe tick infestations and TBDs (7).

**Figure 1:**
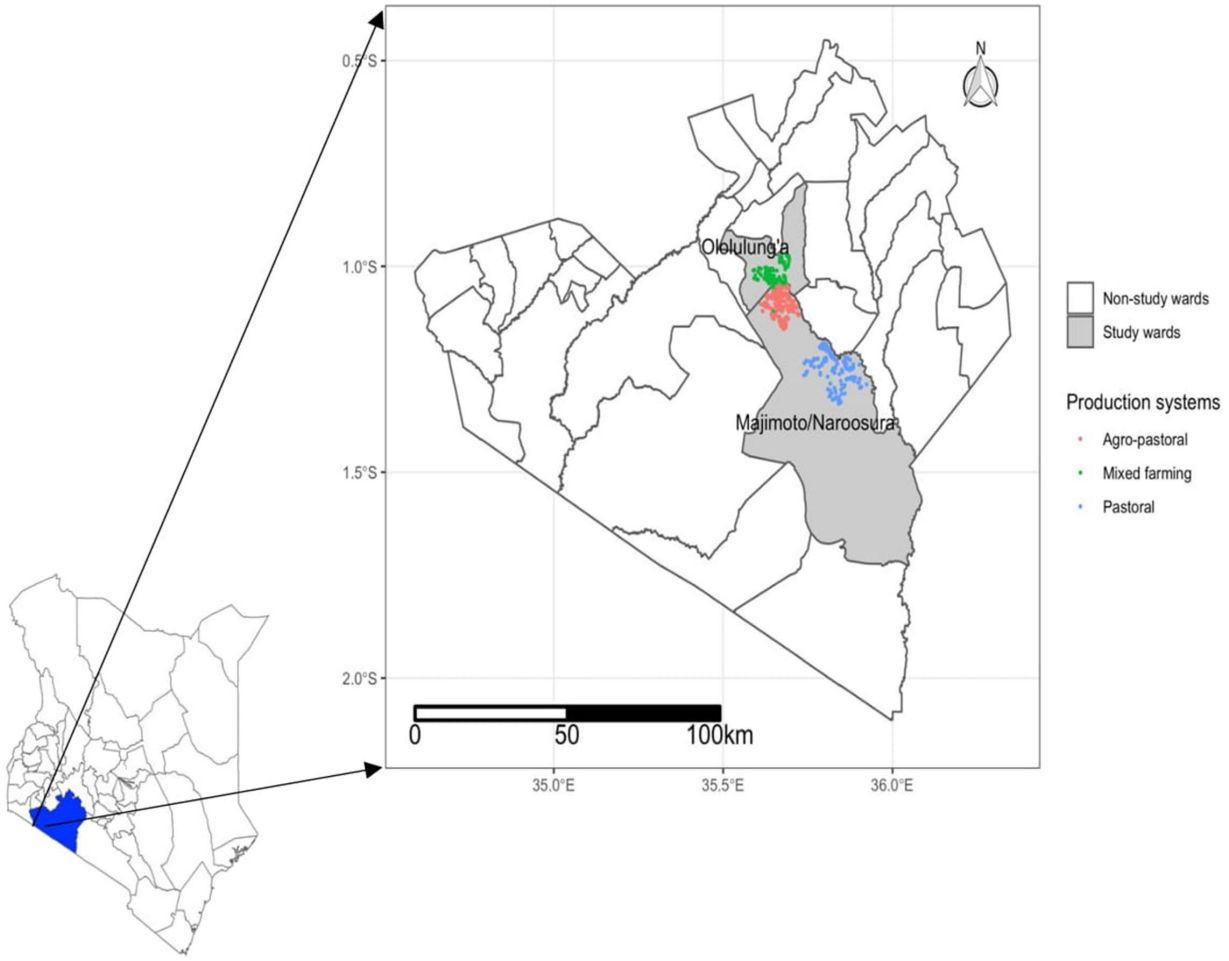
Map of Kenya showing Narok county and specific study wards, the different production system, and locations of the households where this study carried out. Source: the map was generated GPS coordinates captured during the data collection process.

Specifically, two wards in the sub-county, namely Naroosura Maji-Moto and Olololung’a ward, were selected (Figure 1). These wards represent different livestock production systems; mixed farming, agro-pastoral, and pastoral production systems. The selection of the three production systems was based on previous studies that have shown that agroecological conditions, livestock production systems, and farm management practices as primarily the factors associated with the epidemiology of ECF. Specifically, morbidity and mortality rates associated with ECF have been found to vary based on these factors (7, 21). Furthermore, the inclusion of these diverse production systems allowed for a comprehensive exploration of the different EMs within a dynamic interplay of mixed farming, agro-pastoral and pastoral production systems. By examining these production systems, the study aimed to capture the varying beliefs, perceptions and practices that influence ECF control. This holistic approach ensured that the study considers the biological and environmental factors and the cultural and experiential aspects of ECF management among livestock keepers. The livestock keepers were selected from the twelve villages (Table 1) represented in the two wards. Additionally, the study maintained a distance of more than five kilometres between production systems to minimize the likelihood of mixing.

**Table 1:** Production systems and corresponding villages.

Production system	Villages
Agro pastoral	Oloongila, Oloenae, Olkiriaine, Illadoru
Pastoral	Oldonyo-Orasha, Oloornga’nayio, Erupata, Inkasuriak, Nkintintini
Mixed farmers	Olepolos, Masaantare, Olgilai

The Maa community predominantly occupies the three production systems. However, their dependence on livestock for their livelihoods and livestock management practices varies. The Maa people typically reside in homesteads near their cattle pens, enabling them to closely monitor and ensure their herds well-being. This proximity is not solely a practical arrangement. Still, it is deeply woven into their cultural identity, with the belief that the prosperity of the Maa is intricately linked to the well-being of their livestock. In their livestock care practices, the Maa employ a combination of traditional and modern methods. While they have integrated some modern veterinary practices, they still rely on the traditional knowledge passed down through generations. The study population consisted of male livestock keepers aged 18 years and above in different villages. The choice of male participants was based on the socio-cultural context of the community, where the gender roles assigned to men typically involve livestock health and management. Given this socio-cultural context, male participants were better placed to provide information on livestock health and management within the community.

### 2.2 Sampling and ethical clearance

This study employed an ethnographic approach to understand the EMs across the three livestock production systems. This approach focused on capturing the emic perspectives of livestock keepers- their beliefs, perceptions and health-seeking patterns. It was appropriate for understanding the unique worldviews and interpretations of health-related events and behaviors within the specific context of ECF. Purposive sampling was used to select study participants. This sampling approach was chosen deliberately to ensure the inclusion of individuals who could provide the most relevant and insightful information within the specific context of our study objectives.

The study received approvals from the Kenya Medical Research Institute (KEMRI) and the National Council of Science, Technology, and Innovation (NACOSTI) clearance referenced KEMRI/RD/22 and NACOSTI/P/23/23342, respectively. The researchers also shared information about the project with community stakeholders, including its objectives, potential participants, anticipated risks and benefits, confidentiality, anonymity, and voluntary participation. All questions were answered before participants’ written informed consent was sought.

### 2.3 Data collection

Data was collected between April 2023 to January 2024 using multiple data collection methods, including participant observations, in-depth interviews (IDIs), focus group discussions (FGDs), and key informant interviews (KIIs). These methods were triangulated to ensure the validity and reliability of the findings through multiple perspectives and data sources. The IDIs and FGD guides had open-ended questions that served as discussion prompts and guides. The development of the guides was based on the domains of the EM as identified by Kleinman including the definition of illness, etiology, the onset of signs, course of sickness progression and treatment (16). FGDs targeting men were conducted with one hundred participants in total. An additional thirty IDIs were undertaken to provide more comprehensive, in-depth, and varied data. Twenty-five KIIs were also conducted with chiefs, local village elders, animal health care service providers (county and private), and animal health service providers in agro vets across the different wards.

Participant observation was conducted during formal interviews and informal interactions with community members, primarily focusing on men, to help make general observations about the livestock keepers and their animal health-seeking behavior. Observations were made in several settings: the homestead of study participants, livestock trading centers, local agricultural, veterinary shops (agro-vet) and village meetings. The men were selected for these observations due to their predominant role in livestock health management within their community.

Village elders from each selected village helped mobilise participants drawn from different villages. Since this is largely a qualitative study, data saturation was used to assess the adequacy of purposive samples (22).

Six trained researchers, fluent in Swahili and Maasai languages, were involved in the data collection. The interviews were conducted in Maasai or Swahili, depending on the participants’ preferred language. All interviews were audio recorded, with handwritten notes as backup. Additionally, field notes were used to record detailed descriptions of activities, events, interactions and settings.

### 2.4 Data management and analysis

The recorded audio was transcribed and translated into English by research assistants fluent in local and English. The transcripts were reviewed by cross-referencing scripts with interview notes to ensure accuracy and consistency.

The data was entered into QSRNVIVO version 12.5.0 (NVivo qualitative data analysis software; QSR International Pty Ltd. Version 12, 2018) for the development of codes. This allowed the researchers to manage the data, organize ideas, define themes, and draw conclusions iteratively and collaboratively (22, 23). The data were organized and grouped into codes that represented different themes regarding the etiology of ECF, signs of ECF and animal health-seeking behavior. This process was iterative, supported by existing literature, and the modification of themes, enabling the identification of relationships between them identified. The emerging patterns were identified (23). Field notes from participant observations were used to provide context and guide the interpretation of the findings. Translated verbatim quotes have been used to illustrate the key points derived from the study.

## 3 Results

### 3.1 Demographic characteristics

The results are presented and organized to explore the broader domains of EMs as outlined by Kleinman. The results are structured into; local terminology and beliefs associated with ECF, perceived causes, perceived signs of ECF and animal health-seeking behavior. Summaries of each domain and theme are presented in Table 2. A total of 130 participants from twelve villages in Narok South Ward participated in the study. More than a quarter (28%) of the participants were between the ages of 26 and 33, and almost half had no formal education (Table 3).

**Table 2:** Summary of themes and sub-themes for each domain.

Domain	Themes	Sub-themes
Local terminology and beliefs associated with ECF	Meanings associated with the terminology for ECF	Variations in local name for ECF across production systems
	Beliefs about ECF manifestation in different livestock	Parallels between ECF manifestation in different livestock
Perceived causes	Biological causes	Presence of vectors (ticks, mosquitoes, tsetse flies)
	Environmental and ecological contexts contributing to ECF outbreaks	Wildlife-livestock interactions
		Pasture quality and water sources
		Climate and seasonal patterns
Perceived signs	Diagnostic practices of ECF	Traditional diagnostic methods
Animal health seeking behavior	Treatment practices	Self-treatment vs. formal treatment
	Access to veterinary services	Barriers to accessing veterinary services, and decision-making in seeking treatment

**Table 3:** Socio-demographic characteristics of the participants.

Age	18–25 years	23%
	26–33 years	28%
	34–41 years	18%
	42–49 years	10%
	>50 years	20%
Education	None	46%
	Primary	26%
	Secondary	20%
	Tertiary	6%
Marital Status	Married	94%
	Single	6%
	Separated	–
Religion	Traditional	12%
	Christian	87%

### 3.2 Local terminology and beliefs associated with ECF

The local names for ECF varied across different livestock production systems. In certain local contexts, most pastoral production systems commonly referred to ECF as *Oltikana* from the word “*Ntikan,*” which translates as parotid lymph nodes. On the other hand, in the agro-pastoral and mixed production systems, ECF was known as *malaria ya ng’ombe* (cattle malaria). Some livestock keepers in the agro-pastoral and mixed production systems used both terms interchangeably, indicating a fluidity in the local disease terminology.

Across the three production systems, the participants perceived that while ECF could affect all cattle, in some instances, calves were perceived to be at a higher risk compared to larger cattle, as illustrated by these quotes:

ECF affects cattle. However, calves are mainly at a higher risk of death as compared to larger cattle (FGD Pastoral).

Sometimes, cattle are affected by ECF, but they recover quickly. However, this is different for calves. Ten of my neighbor’s calves died last year due to ECF (FGD Agro-pastoral)

In the agro-pastoral and pastoral production systems, sheep were also perceived to be prone to ECF. One participant remarked.

During the dry season, that is when Oltikana infects cattle and also sheep (IDI Pastoral)

However, key informants clarified that this belief stemmed from a common misunderstanding among livestock keepers. Key informants indicated that the disease affecting sheep was a different condition that presented similar signs to ECF leading to confusion:

We have the Nairobi sheep disease (NSD) in sheep, which the locals mostly confuse with ECF in cattle. It is very rampant here and typically presents as high fever, loss of appetite, nasal discharge, diarrhea, and inflammation. The fact that the ticks cause the disease creates the perception among the locals that it is ECF (KII-Animal Health Officer-Ewaso Ng’iro)

Most livestock keepers will come and tell you my sheep look like they have been infected by malaria. When sheep overeat, they might start breathing heavily, have bloody diarrhea, and appear dull; signs locals perceive to be those of ECF (KII Agro-vet Ololulung’a)

Few livestock keepers (4 IDIs) in the agro-pastoral and mixed farmers production system perceived that *Oltikana* also affected human beings, as in these excerpts:

We also call Oltikana Malaria, and it affects both man and animals. With the animals, it affects their mouth and limbs (IDI Agro-Pastoral)

Oltikana affects the cattle and also the people (IDI Mixed Farmers)

From the information that I have gotten after going to the hospitals, it is caused by mosquitoes. When the mosquitoes breed in stagnant water and bite you, they cause malaria. We are advised to sleep under the mosquito nets, and I tell my wife at night to close the door and the windows so that the mosquitoes will not enter my house and cause malaria to my children. So, it is caused by that mosquito. When we go to the local clinic, we are told we have Oltikana caused by mosquitos, and then they give us the medicines (IDI Agro-Pastoral)

### 3.3 Perceived causes of ECF

While livestock keepers across the three production systems commonly believed that ticks contributed to the occurrence of ECF, it was evident that there was no single cause. In addition to the presence of ticks being linked to the occurrence of ECF, various other factors were also linked to ECF as presented in Table 4.

**Table 4:** Perceived causes of ECF in cattle by livestock keepers.

Perceived causes of ECF	Quotes
Tsetse flies	*When the cattle are bitten by some fly called orkimpai(tse tse fly), the cattle start to develop ECF signs (IDI Pastoral)*
Mosquitos	*When the cattle go to drink the water, the mosquitos that are usually found in water sting them, and this causes ECF (IDI Agro-Pastoral)*
Stagnant, contaminated, and dirty water	*When cattle drink the stagnant water contaminated by the leaves of olerai(acacia), they will be infected with ECF (FGD Agro-Pastoral)*
Common grazing and watering sites	*Healthy cattle may drink water together with infected cows, thus transmission of ECF to the healthy cows (FGD Agro-pastoral)*
Wildlife	*When the cattle eat grass from the areas where the wildebeests have given birth, they may be infected with ECF. They may also be infected with it if they are grazing together and their skin or fur come in contact with each other then they will be infected with it (IDI Pastoral)*
Green grass and leaves	*It is caused by green leaves and grass, especially around the forest area (IDI-Agro pastoral)*
Birds	*Sometimes, there are migratory birds that feed on the limbs and the back of the cow and transmit many diseases from other areas, including ECF (IDI Pastoral)*

However, certain causation factors were specific to the production system. In agro-pastoral and mixed farming systems, mosquitoes were frequently cited as a cause of ECF. This perception was less common in pastoral systems. In contrast, migratory birds were predominantly mentioned in pastoral systems as possible transmitters for ECF, as in the excerpt;

The bird will contribute to the transmission of the diseases because even if they feed on one cattle when they feed to the next cattle, they will transmit the disease (IDI Pastoral)

There were also diverse and contrasting perceptions regarding the role of wildlife in the causation of ECF. While most livestock keepers across the different production systems believed that wildlife was responsible for ECF outbreaks, participants from the pastoral production system (2 out of the 18 FGDs) believed that the observed signs indicated a different condition. This quote exemplifies this:

There are months when the wildebeests give birth to the calves. When they do so, and the cattle eat the grass from the region where it had given birth, the cattle will be infected with engati (Malignant Catarrhal Fever MCF) (FGD Pastoral)

When cattle graze where wildebeests have birthed, they are not infected with Oltikana; they are infected with another disease that affects the eyes and is incurable (FGD Pastoral)

Local signs associated with ECF.

Livestock keepers across the different production systems diverse range of signs associated with ECF, as detailed in Table 5. These observations, rooted in their local knowledge and direct experiences, offer a unique perspective on the multifaceted nature of ECF manifestations.

**Table 5:** Livestock keepers reported clinical signs associated with ECF in cattle.

Clinical sign	Participant descriptions	Quotes
Excessive salivation	Excessive drooling or foaming from the mouth	*Some will salivate a lot while some will produce a lot of froth (FGD Agro-Pastoral)*
Nasal discharge	Heavy mucus or green mucus	*When they are infected, they will produce thick green mucus (IDI Mixed Farmers)*
Dry muzzle	Cracked muzzle, sometimes bleeding	*In the first stages of infection, their nose will dry up and appear cracked. You will also see flies on the nose (FGD Agro-pastoral)*
Corneal Opacity	Change in eye colour (red, brown, white, blue) or sometimes blind	*The eye appears whitish, and sometimes the disease may result in blindness (FGD-Pastoral)*
Lacrimation	The cattle appears to be crying	*The most obvious symptom is reflected in the cattle’s eyes. The eyes start shedding tears (FGD Mixed Farmers)*
Diarrhoea	Watery and sometimes bloody stool	*Diarrhea indicates that the cattle have been affected by the disease and they may die after two days of producing diarrheal that is bloody. We usually call it Sertet (IDI Pastoral)*
Fever	Shivering	*You will also notice that the cattle are shivering (ID Agro-pastoral)*
Rough hair coat	Hair appears to stand	*I usually observe that the cattle have their hair standing (IDI Mixed Farmers)*
Loss of appetite	Decreased appetite, reluctance to feed and loss of weight	*The cattle will have no appetite, they will significantly lose weight, and will also be weak (IDI Pastoral)*
Depression	Standing alone or looking inactive	*They always stand alone, have their head down, and they will always stand under the shade, and look inactive (FGD Pastoral)*
Cough	Cough, difficulty breathing	*There is a type of ECF that makes the cattle cough and sometimes makes it difficult for cattle to breathe (IDI Agro-pastoral)*
Constipation	Hard and sometimes bloody stool	*You will find it removing something that is hard and has some red patches (FGD Mixed Farmers)*
Abortion	Giving birth before the time	*The cow gives birth before the pregnancy matures, and when the pregnancy matures, the cow would still have unusual signs (FGD Mixed Farmers)*

Participants also observed different manifestations of ECF that were differentiated by their presented signs and severity. Variations included ECF that affected eyes, intestines and damaged liver. In terms of severity, some variations could be treated with antibiotics; others were acute in nature.

There are different types of Oltikana. There is one that affects the intestines, one that affects the eyes, and the other causes swelling of the lymph nodes (FGD Agro Pastoralists)

There is a type of Oltikana that the cattle are infected with, making them produce bloody cow dung. This shows that the cows have been affected by the disease and may die two days after producing the bloody cows’ dung. We usually call it Sertet. This results in faster death of the cows. There is also another type of Oltikana that causes the cows to have diarrhea which is usually manageable. We usually say that the cows have Mbinik; this is when they have drunk contaminated water (IDI Pastoralists)

However, KII informants noted that these manifestations did not indicate ECF but other livestock diseases, as illustrated in this quote.

In some areas, the leaves of specific trees fall into the water; livestock keepers believe that the water will develop some taste due to these leaves. So, if animals drink that water, they will become sick. Livestock keepers believe that it is also ECF because clinical signs of that disease resemble ECF, so they call it ECF. So, where the water is there, you can find farmers saying the animals are suffering from ECF due to the stagnant water (KII Vet Ololulung’a)

Most of the livestock keepers do not understand the signs of cattle infected with ECF. So, they may state the signs of MCF because they cannot differentiate it from Oltikana (KII Animal Health Practioner Ololung’a)

### 3.4 Animal health-seeking behavior

In identified ECF cases, livestock keepers resorted to various treatment practices. These ranged from self-treatment to seeking veterinary assistance and selling the affected cattle. Self-treatment was often the initial choice for livestock keepers when dealing with ECF cases. Livestock keepers would often purchase medicine from the local agricultural, veterinary shops (agro-vet) or medications already existing in the household:

I buy Terramycin and treat the cows when they are infected with Oltikana (IDI Pastoral)

Every homestead here typically has some drugs in the houses, mostly terramycin. The moment they see an animal suffering, they do not even care which disease it is; they automatically bring their oxytetracycline and inject that animal (KII Vet Ololulung’a)

If the initial attempts at self-treatment failed, livestock keepers would consider seeking what they perceived to be “stronger” drugs. This would either be a higher dosage than the initial drug or buying an entirely different drug, as demonstrated by these quotes:

When the cows are infected with ECF, we usually administer 10% Terramycin or Penicillin, and some administer 20% Terramycin. If they are not effective, then we administer the 30% (FGD Agro-pastoral)

When the cows are infected with Oltikana, I will administer Terramycin. When the cows do not get better after two or three days, I will administer Penicillin (FGD Pastoralists)

Key informants attributed this to the varied treatment practices to costs, as exemplified in the quote:

We usually recommend Terramycin 30% because of its effectiveness. However, this is expensive for the livestock keepers, and that is why they often go for the 10% because it is affordable (KII Agro-Vet Ewaso Ng’iro)

As a standard practice where self-treatment did not yield the desired result, livestock keepers often inquire about treatments employed by family/peers in response to similar situations. These discussions focused on identifying potentially effective drugs and their sources obtained (whether from a fellow peer or agro vet shops) as exemplified in this quote:

I had given up on treating my cattle, and my neighbor recommended that I administer Butalex. This was very effective (FGD- Pastoral)

Livestock keepers often get information from their peers, and then afterwards, they will go to the agro vet shops and purchase the drug (KII Agro-Vet Ololulung’a)

Veterinary doctors were viewed as the last resort for livestock keepers if self-treatment failed. Several factors influenced the decision to have veterinary doctors as the last option, as detailed in Table 6.

**Table 6:** Reasons for livestock keepers’ reluctance to consult animal health practitioners.

Lack of trust	*We do not trust these doctors; most just come to market their products, which sometimes do not work (FGD Agro-pastoral)*
Cost considerations	*If I consult a veterinary doctor, I must pay for their transport and services, which is very costly. (FGD-Mixed Farmers)*
Experience with livestock	*We are the first doctors because we were raised with livestock and learned from our fathers. We can tell when our livestock are sick and how to treat them (FGD Pastoral)* *Some say that they know more than the veterinary doctors because they are older and have more experience in taking care of the cows than the veterinary doctors (KII Ololulung’a)*
Selective veterinary consultations	*During an outbreak of the disease, that is the time that we call the veterinary doctors for vaccination. Recently, we had our livestock vaccinated because it was announced that our neighbours had experienced a Foot and Mouth Disease (FMD) outbreak (FGD Pastoral)*
Uncertain treatment outcomes	*There are times when even the veterinary doctor comes to treat the cows, and they still do not get cured of the disease (FGD Agro-pastoral)*
Inaccessibility	*It will take a long time before accessing a doctor since they are few and have to serve many farmers (FGD Agro-pastoral)*

In cases where livestock keepers chose not to consult a veterinarian after self-treatment proved ineffective, another standard course of action was to sell the affected cattle. This was to minimize losses and lessen the financial impact of an unsuccessful treatment.

If the cattle do not respond to treatment, the best option is to sell at a loss and get money which you can even use to purchase sheep or other household items (FGD Pastoral)

If there are no changes and the cattle are still sick, I take them to the slaughterhouse (IDI pastoral)

Livestock keepers’ perceptions about the causes of ECF and lived experiences significantly shaped their approach to the prevention measures. Preventive measures reported were mainly spraying or injection to control ticks, fencing of land, and avoidance of stagnant water, as presented in Table 7.

**Table 7:** ECF prevention measures.

Preventive measures	Quotes
Tick Control	*We have created a timetable that we wash our cows every Friday or Saturday because when we control the ticks, we control the disease (IDI Agro-pastoral)* *We wash and inject the cattle to prevent the ticks (IDI Mixed Farmers)*
Fencing	*Previously, the livestock keepers had not fenced their farms so the cows usually grazed together. Currently, the livestock keepers have fenced the land, and this resulted in a lower rate of infection of Oltikana (IDI Agro-pastoral)* *We have fenced our farms so that the cows cannot graze beyond other farms and also to prevent the wild animals from entering the farms and mixing with the cows (FGD pastoral)*
Avoidance of migration to certain areas	*Some of the livestock keepers from this area have gone to Mara, and they have slaughtered three cows, some five cows, because they were infected with ECF. So, we hardly go to that area to graze our cows even if it rains in that area (FGD Agro-pastoralists)* *We avoid going to the Mau region because cattle get ECF due to the cold weather and the green leaves and grass from the forest, which the cattle are not adapted to (FGD Pastoralists)*
Avoidance of stagnant water	*We prevent our cattle from drinking stagnant or contaminated water since they may be infected with ECF (FGD Mixed Farmers)*

However, there were varied responses among the livestock keepers. Some measures were not considered adequate,

There is a specific tick that is resistant to acaricides. You may spray the cows, and after a few days, you find that the number of ticks has increased (FGD Mixed Farmers)

When the dry season begins in June, ticks begin infesting the cows, but they primarily affect the cows in October, November, December, and late January. During this time, the ticks also resist the acaricide unless you spray the cattle immediately after seeing the ticks. Even if you set a timetable for spraying your cows every week, they will still develop a resistance to the acaricide (IDI Agro-Pastoral)

The brown tick is the deadliest and usually affects the thin cows. It is also resistant to acaricides, and it mainly affects the cows when they are thin acaricide (IDI Pastoral)

In other cases, some prevention measures were also impractical, particularly during the drought period when livestock keepers had to make the difficult decision of either migrating and risking disease exposure or losing their cattle to hunger due to lack of water and pasture. This is exemplified in the following quotes:

In the dry season, it is tough to control the cows from interacting with each other because, at that time, we have to look for pasture and water (FGD Agro pastoral)

I would rather my cattle die from ECF than of hunger because if they are hungry, all will die, but with ECF, only very few cattle will be infected, and this can be easily treated (FGD Pastoral)

Notably, vaccination did not emerge as a prevention measure for ECF. When asked about vaccination, most of the FGDs and IDIs revealed that livestock keepers were not aware of an existing vaccine for ECF.

I have not heard of the ECF vaccine; I know the medicine used when the animal is sick. What can prevent ECF is frequent spraying of the cattle to ensure that they do not have ticks (FGD Agro-Pastoral)

There is no vaccine completely for Oltikana. It has never been heard (FGD Mixed Farmers)

I have only heard of the medicine that is used to treat Oltikana (IDI Pastoral)

However, there was a contrast in awareness in different regions. In one FGD conducted in the pastoral region and 2 IDIs from the same area, participants mentioned familiarity with the vaccine and information they acquired from livestock keepers in areas where ECF was perceived to be severe. This is exemplified in the following quotes:

It is available but costly, around 2000kshs for one cattle (FGD Pastoral)

I have seen a friend in Lemek using it. It is injected in the ntikan (parotid lymph nodes) but is quite expensive (IDI Pastoral)

## 4 Discussion

This study utilized Kleinman’s explanatory model to understand the etiology of ECF, signs of ECF and animal health-seeking behavior of livestock keepers across different livestock production systems (pastoral, agro-pastoral and mixed farmers). Across the different production systems, various names were attributed to ECF, reflecting both the body parts affected or the signs the animal presented and the perceived risk factors. In the pastoral production system, the local name *Oltikana* was mainly linked to observable signs, or the organ system affected, which is “Ntikan” which translates as parotid lymph nodes, revealing that ECF affected the lymph nodes of the cattle. Similarly, *malaria ya ng’ombe* (cattle malaria), as commonly used in the agropastoral and mixed farming production system, derived its name from the belief among livestock keepers that mosquitoes were risk factors for ECF, thereby associating the disease with malaria. These findings were consistent with other studies that emphasized the prevalence of local terms for describing livestock diseases that were mainly based on signs of the disease in both live animals and carcasses, contact with risk factors, seasonality or location of outbreaks, species, and age of the affected animal (24–26). In the context of this study, the observed variability in local disease terminology could be explained by environmental differences where each production system may prioritise different aspects of the disease based on their specific experiences and observations. Furthermore, social interactions among these various groups could explain why livestock keepers in the agro-pastoral and mixed farming production systems use the term interchangeably. Livestock keepers observed that cattle could contract ECF and noted a significant difference in susceptibility between calves and adult cattle. Across the three livestock production systems, calves were perceived to be more vulnerable to ECF, as observed by livestock keepers, who found the disease much more severe in calves than in adult cattle. These observations contradict findings by Chenyambuga et al. (13) and Chenais and Fischer (27), which indicate that livestock keepers perceived that calves only could contract ECF. In addition, livestock keepers also perceived that sheep could contract ECF, a perception that was particularly prevalent in the pastoral and agropastoral systems. This belief stemmed from the significant similarities between the symptoms of ECF in cattle and sheep. In addition, ticks being a risk factor strengthened this belief. Among the agro-pastoral and mixed farmers, ECF was also thought to affect human beings because mosquitos were thought to be among the risk factors. Similarly, another study conducted among pastoralists in Northern and Eastern Tanzania demonstrated that symptoms of certain diseases in livestock were compared to those in humans. Pastoralists observed similarities between foot and mouth (FMD) in cattle and humans such as blisters around the mouth, nasal discharge, flu-like symptoms and cough. Moreover, the occurrences of these illnesses in humans coincided with the outbreaks of FMD in cattle, suggesting a potential link (28). These findings highlight the importance of understanding local perceptions and terminologies in addressing livestock diseases. Recognising factors influencing the disease’s terminologies can improve communication and intervention strategies within different contexts.

Livestock keepers across the three production systems attributed ticks to ECF consistent with the scientific understanding that it is transmitted by the *Theileria parva* parasite found on ticks (7). In addition to ticks, tsetse flies, mosquitos, stagnant or contaminated water, livestock wildlife interactions, green leaves, and birds were also considered risk factors for ECF. These findings are consistent with Inambao (12), Chenais and Fischer (27), and Caudell et al. (29) where flies, green grass, and water were perceived to cause ECF. Among agropastoral and mixed farming production systems, mosquitos and stagnant or contaminated water were perceived to be a risk factor for ECF. The association of ECF with mosquitos and stagnant water was shaped by observations in their environment. This may have also been influenced by previous circumstances, possibly coinciding with an outbreak of malaria among human populations or an incidence of livestock diseases (not necessarily ECF) forming a basis of a local understanding of disease transmission. Stagnant water and mosquitos, therefore, were common factors for both malaria in humans and ECF in cattle, leading to the perception that they were linked. Since pastoralists did not attribute ECF to mosquitoes, their understanding and experience of water as a contributing factor to the disease varied. In the pastoral production system, livestock keepers perceived that cattle often got ECF after drinking from stagnant or contaminated water, especially during or after dry seasons. Observational experiences played a significant role in shaping the understanding of risk across the different production systems. Understanding these observational experiences is crucial for designing effective interventions. Interventions that take into account the community’s experiences could be more culturally sensitive and effective as interventions can be designed to address the misconceptions that stem from these observations.

Local names given to ECF were descriptive and related directly to what was observed in the livestock. In addition to observable signs, the livestock keepers incorporated various contextual factors into their disease naming and diagnostic procedures. Similarly, other studies conducted among pastoralist communities show that understanding symptoms of the diseases, knowledge of vectors, seasonal outbreaks of diseases and age and species affected by diseases were important tools for traditional disease diagnostic procedures (24–26, 30). These diagnostic procedures were holistic, combining observable symptoms and other contextual factors that enabled livestock keepers to differentiate between different diseases effectively. Among the agro-pastoralists and mixed farmers, ECF was diagnosed by observing the presence of vectors such as ticks, tsetse flies and mosquitoes in addition to observing the environment for stagnant water, and the presence of green grass and observing seasonal and migratory patterns. Pastoralists also diagnosed ECF by observing the presence of vectors such as ticks and the environment for migratory birds as well as observing seasonal and migratory patterns. Their diagnostic procedures were grounded in extensive traditional knowledge and environmental observations. However, despite their diagnostic procedures, the study showed that most livestock keepers faced challenges distinguishing between diseases, particularly MCF. Several studies have demonstrated that ECF is often confused with other diseases such as anaplasma, babesiosis, cowdriasis, and MCF (30–33). Further in this study, livestock keepers perceived different forms of variants of ECF that not only manifest differently in livestock but also had varying levels of severity. These forms of ECF were perceived by livestock keepers to affect different parts of the body. For example, there was the ECF variant that caused, the enlargement of the spleen, the swelling of the lymph nodes, the shedding of tears, coughing, bloody diarrhoea and constipation. However, from a biomedical perspective, some of these signs such as swelling of lymph nodes are early indicators of the disease. Other signs such as lacrimation (shedding of tears), constipation, bloody diarrhoea and respiratory distress appear as the disease progresses (34). Hence, what livestock keepers perceive as different forms of ECF are different stages of ECF progression. Although livestock keepers lacked the convectional biomedical knowledge their extensive traditional knowledge and practical experience enable livestock keepers to manage livestock health.

Livestock keepers across the three production systems often resorted to self-treatment as the initial response in suspected cases of ECF. In most instances, the drugs were readily available in the household, having been purchased in advance from the local agro-vet shops. Oxytetracycline injections (OTC) of 10 to 20% were used in most cases as the first course of treatment; however, in cases where treatment failed, either a higher concentration (OTC 30% was used) or other drugs such as Penicillin and streptomycin (Penstrep) and Butalex (Buparvaquone Injection). Similar findings reported routine use of injectable oxytetracycline at 10% among pastoralist communities, with an increase in dosages where treatment proved ineffective (26, 29). Further findings indicate that peers, families, and neighbors were also involved in livestock health management by giving advice or recommendations on potentially effective drugs from agro-vet shops when treatment failed. Agro-vet shops, serving as significant sources for procuring veterinary drugs within the community, played a crucial role in livestock health management. A study among agro-pastoralists in Northern Tanzania found that these shops served as important sources of veterinary drugs rather than for advice (26). Although livestock keepers often drew on their extensive generational knowledge, observational skills and cultural experiences as livestock experts, this did not diminish the feelings among most that they resorted to self-treatment due to the unavailability or high costs of engaging animal health practitioners. Generally, livestock keepers described these services as being distant, expensive or inaccessible. Trust was also connected to the livestock keeper’s belief in the animal health practitioner’s competence (capabilities, knowledge and skills) or their motivations (business-oriented rather than desire to help). Furthermore, the lack of responsiveness when contacted also contributed to the livestock keeper’s level of trust. Animal health-seeking behavior did not only depend on traditional knowledge of the livestock keepers but also on their ability to make choices within the broader context in which they lived. Several studies have also emphasized the importance of understanding context and its influence on health-seeking behaviors (26, 35, 36). Efforts to enhance livestock health should take into account the complex factors influencing health-seeking behavior. This holistic approach ensures that interventions are both empowering and feasible, ultimately leading to improved health outcomes for livestock.

Livestock keepers’ holistic approach to disease diagnosis not only enhanced their ability to identify livestock diseases and treat livestock diseases but also informed their preventive measures. Although most livestock keepers were unaware of ITM, they took precautions based on their indigenous knowledge of the ECF’s course and vector. Therefore, the use of acaricides to control ticks, fencing, avoidance of stagnant water, interaction of wildlife and livestock and sometimes avoidance of locations that were perceived to be ECF prevalent were some of the precautions livestock keepers practised. Through observing their environment, experience and community-based knowledge sharing, livestock keepers developed these strategies to protect their livestock from ECF. Similarly, a study among the Maasai pastoralists demonstrated that since MCF had no cure, livestock keepers deliberately kept their cattle away from wildebeests during calving seasons as wildebeests were considered the silent carriers for the MCF (30). However, in this study, livestock keepers did not view some of the precautionary measures as very effective due to the resistance of specific ticks to acaricides. Additionally, fencing and avoidance of wildlife-livestock interaction was not always practical, especially during periods of drought when livestock keepers had to migrate in search of water and pasture for their livestock. Understanding livestock keepers’ prevention strategies for diseases is crucial for the development of culturally relevant, effective and sustainable interventions. This ensures that interventions are grounded in local realities, builds trust and cooperation within the community, and leverages existing knowledge and practices to enhance overall disease control efforts.

### 4.1 Limitations of the study

First, this study used qualitative data to understand perceptions of ECF and health-seeking behaviors of livestock keepers across pastoral, agro-pastoral and mixed farming production systems, limiting generalizability. Although the results cannot be generalized, this study provides detailed and context-specific information that can be used to develop tailor-made interventions. Second, the study relied on livestock keepers’ descriptions and interpretation of symptoms without biomedical verification. This could lead to misclassification or misunderstanding of diseases. The study highlights access and trust issues concerning veterinary services but does not explore the systemic issues in depth. Understanding broader structural constraints could provide a more detailed picture. The study recognizes the substantial traditional knowledge of livestock keepers but does not thoroughly evaluate the depth and scope of this knowledge. A more comprehensive investigation could offer valuable insights into the effectiveness and constraints of traditional practices. However, the findings provide a basis on which future studies on ECF can build.

## 5 Conclusions and recommendations

The study delves into livestock keepers’ perceptions and health-seeking behaviors regarding ECF. The study revealed variations in terminology for ECF across different livestock production systems, highlighting the influence of environmental factors on disease nomenclature. Livestock keepers employed holistic approaches to disease diagnosis, combining observable symptoms with contextual factors such as the seasonal effects, knowledge of risk factors or vectors and environmental conditions. This approach highlights the value of traditional knowledge and practices in livestock disease control. To integrate cultural and scientific knowledge, interventions aimed at enhancing livestock health should focus on improving communication between livestock-keeping communities and animal health professionals. This ensures a thorough understanding of ECF and leverages the strength of traditional and biomedical approaches. Developing culturally sensitive educational materials that consider linguistic variations and terminologies used across different production systems is crucial. Additionally, comparative studies should be conducted to assess the effectiveness of traditional diagnostic methods alongside biomedical techniques. These studies identify areas of complementarity and integration between the two approaches, fostering a more integrated and effective livestock health management system.

## Data availability statement

The original contributions presented in the study are included in the article/supplementary material, further inquiries can be directed to the corresponding author.

## Ethics statement

The study was approved by National Council of Science, Technology, and Innovation (NACOSTI), Kenya (NACOSTI/P/23/23342). The studies were conducted in accordance with the local legislation and institutional requirements. The participants provided their written informed consent to participate in this study.

## Author contributions

AM: Conceptualization, Data curation, Formal analysis, Investigation, Methodology, Project administration, Visualization, Writing – original draft. JM: Project administration, Resources, Writing – review & editing. NM: Writing – review & editing. ST: Funding acquisition, Writing – review & editing. SB: Supervision, Validation, Writing – review & editing.
